# Radicular Dentin Thickness and Root Canal Morphology of Mandibular Incisors in Indian Subpopulation Using Cone Beam Computed Tomography

**DOI:** 10.7759/cureus.73355

**Published:** 2024-11-09

**Authors:** Gokul Krishnan, Sudhanva M E., Anithakumari Rangappa, Vikram Rangaswamy, Chethana S Murthy, Naveen Kumar N.

**Affiliations:** 1 Department of Conservative Dentistry and Endodontics, Vokkaligara Sangha Dental College and Hospital, Bengaluru, IND

**Keywords:** ahmed classification, cone-beam computed tomography (cbct), mandibular incisors, radicular dentin thickness, root canal morphology, vertucci classification

## Abstract

Aim

Using two classifications, this study assessed root morphology and canal configuration and measured the Dentin thickness (DT) and canal shapes.

Methods

Cone beam computed tomography (CBCT) with 400 Mandibular Incisors was collected and assessed for the number, length, curvature of roots, number of canals, bifurcation level, configurations based on Vertucci’s and Ahmed’s classification, DT and canal shape at 3, 6, 9 mm from the apex. The collected data was subjected to statistical analysis with a level of significance at p<0.05.

Results

All samples had one root, averaging 12.769 ± 1.128 mm in central incisor (CI) and 13.044 ± 1.235 mm in lateral incisor (LI), with most roots being straight. Most samples had one canal in both teeth, with bifurcations most frequent in the middle third. The most frequent configuration was type 1 Vertucci or ^1^CI^1^/^1^LI^1 ^by Ahmed, followed by type 3 or ^1^CI^1-2-1^/^1^LI^1-2-1^. One sample, not classifiable under Vertucci, was classified as ^1^CI^1-3-1 ^by Ahmed. The mean DT for CI was 3.18 ± 0.639 mm, 3.72 ± 0.671 mm and 4.43 ± 0.754 mm labiolingually and 1.578 ± 0.342 mm, 1.881 ± 0.374 mm, 2.283 ± 0.465 mm mesioditally at 3, 6, 9 mm from the apex, respectively. For LI, mean DT was 3.41 ± 0.916 mm, 3.90 ± 0.702 mm and 4.55 ± 0.746 mm labiolingually and 1.63 ± 0.322 mm, 1.981 ± 0.485 mm, 2.55 ± 0.470 mm mesioditally at 3, 6, 9 mm from the apex respectively, canal shape changed from oval to round, from apical to coronal.

Conclusion

Single canals were the most common, followed by two canals. The middle third of the canal had the most bifurcations. Vertucci type 1 or Ahmed's ^1^CI^1^/^1^LI^1 ^ was the most commonly reported canal configuration, with one sample that could not be classified under Vertucci but could be classified using Ahmed classification. DT increased apical to coronal. The canal shape changed from oval to rounded, from apical to coronal.

## Introduction

Root canal treatment is complex. Each tooth has unique anatomical and morphological features, which can vary between populations, within populations, or even within individuals [1]. Understanding these variations is crucial for successful treatment outcomes. Insufficient knowledge of root canal anatomy and morphology can lead clinicians to overlook canals, particularly in mandibular incisors (MI), where they might mistakenly assume a single canal and miss the second one, potentially resulting in treatment failure. Recent literature reveals significant variations in the anatomy and morphology of MI teeth [2,3].

Root walls have varying Dentin thickness (DT) at different levels. DT is important while planning endodontic therapy as it is crucial in maintaining root strength [4]. MI have roots with mesiodistal (MD) dimensions smaller than the Labiolingual (LL) dimension, hence more are fracture prone. Hence, DT and Canal shape were assessed at different root levels in MI.

Weine et al. first classified various types of canal morphology within a single tooth [5]. Then, the widely adopted Vertucci classification was introduced in 1984 [6]. Despite its popularity, it has limitations in categorizing all reported canal configurations. Ahmed et al. [1] proposed a new classification system based on a coding system, which offers flexibility in naming and assessing any canal configuration. This new system is poised to replace the Vertucci classification in future studies. Hence this system was also considered in this study.

Multiple studies have examined the canal configurations of MI, primarily using the Vertucci classification in the Indian population. Only three studies have used both the Vertucci and Ahmed classifications [7-9], but none focused on the Indian population. This study aims to compare both classifications in the Indian subpopulation and measure DT in MI combining a lesser-studied population with a more sophisticated classification method. Thus, this current study aims to help gain an understanding of the root morphologies, and canal configurations using two classification systems and to measure the DT and Canal shapes at 3, 6, and 9 mm of MI in the Indian sub-population using cone beam computed tomography (CBCT).

## Materials and methods

The study protocol was reviewed and approved by the institutional ethical committee of Kempegowda Institute of Medical Sciences, Bengaluru (Reference number: KIMS/IEC/D022/D/2022). The sample size was calculated to be 400, with a confidence interval set at 95%, so CBCT scans that consisted of 400 MI were collected from CBCT centers in Bengaluru with the patient's consent. The scans were from patients of Indian origin and were not performed specifically for this study. Only scans taken for diagnosis and treatment planning were used. Personal details, including age and sex, were recorded and kept confidential, accessible only to the primary investigator. CBCT scans of healthy, fully developed MI from individuals above 20 years old of Indian origin were included in the study, and scans with MI that had preexisting restorations, treatments, pathologies, or artefacts were excluded from the study.

Image acquisition and evaluation parameters

The current study involves retrospective analysis; hence, no new scans were taken; all scans were obtained from the archives of a CBCT center. CBCT scans were acquired using the Kodak 9300 3D Imaging System (Eastman Kodak Company, Rochester, NY). The scanning parameters were 90-100 kV, 4-6 mA, or 2-6 seconds, with a voxel size of 0.180 mm, slice thickness of 1.0 mm, and a field of view (FOV) of 10 cm x 10 cm, 10 cm x 5 cm, or 5 cm x 5 cm. The acquisition followed the manufacturer's recommended protocol and was supervised by an experienced radiologist. The CBCT volume data was reconstructed using CS 3D Imaging Software Version 3.2.9 (Carestream Health Inc., Rochester, NY), with contrast and brightness adjusted for optimal visualization using the software’s image-processing tools.

**Figure 1:**
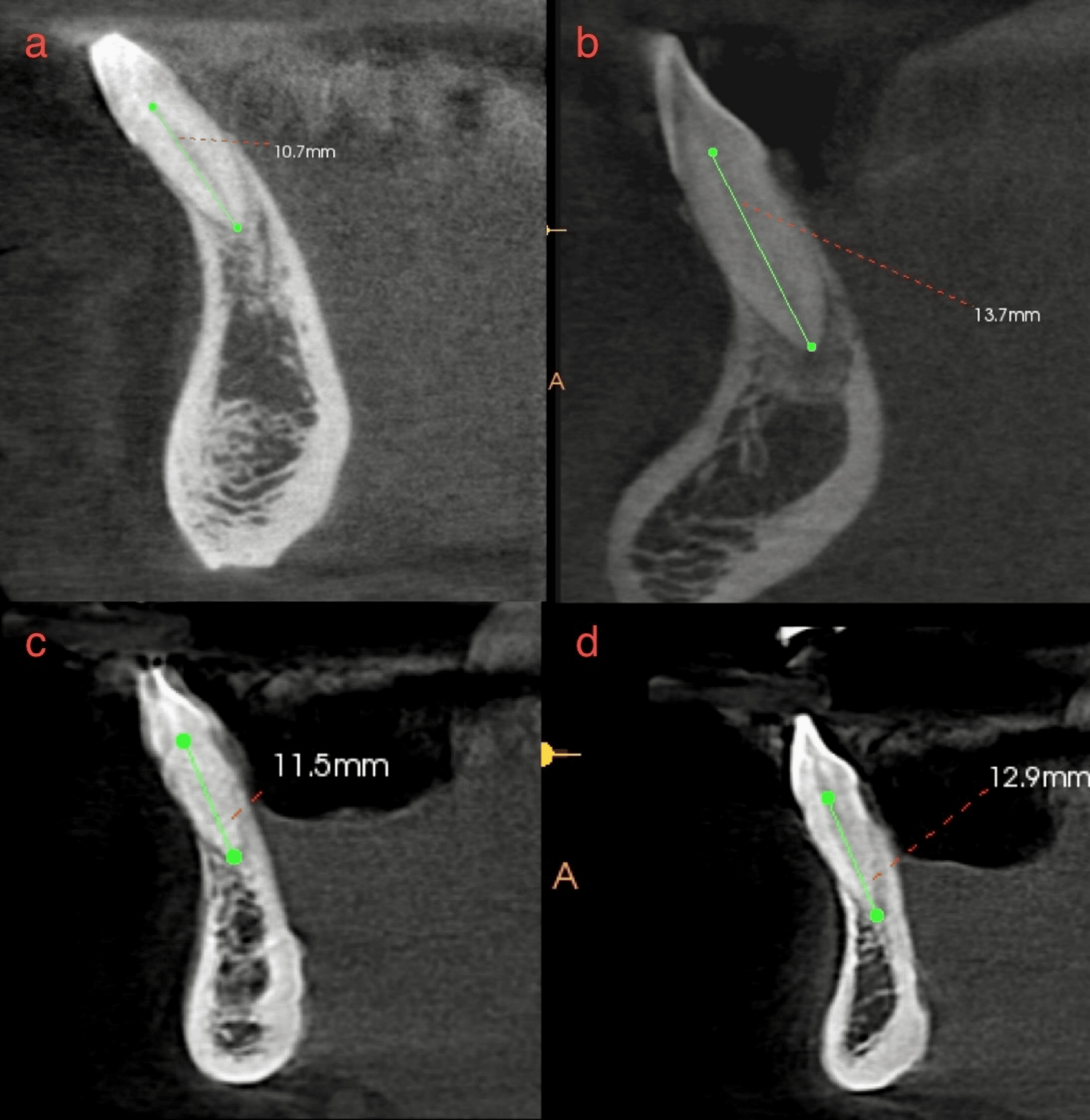
Multiple CBCT sections that illustrate the method to determine root length. (a-d) Four different scans depicting root length measurement from the cementoenamel junction to the apex. CBCT - Cone beam computed tomography

**Figure 2:**
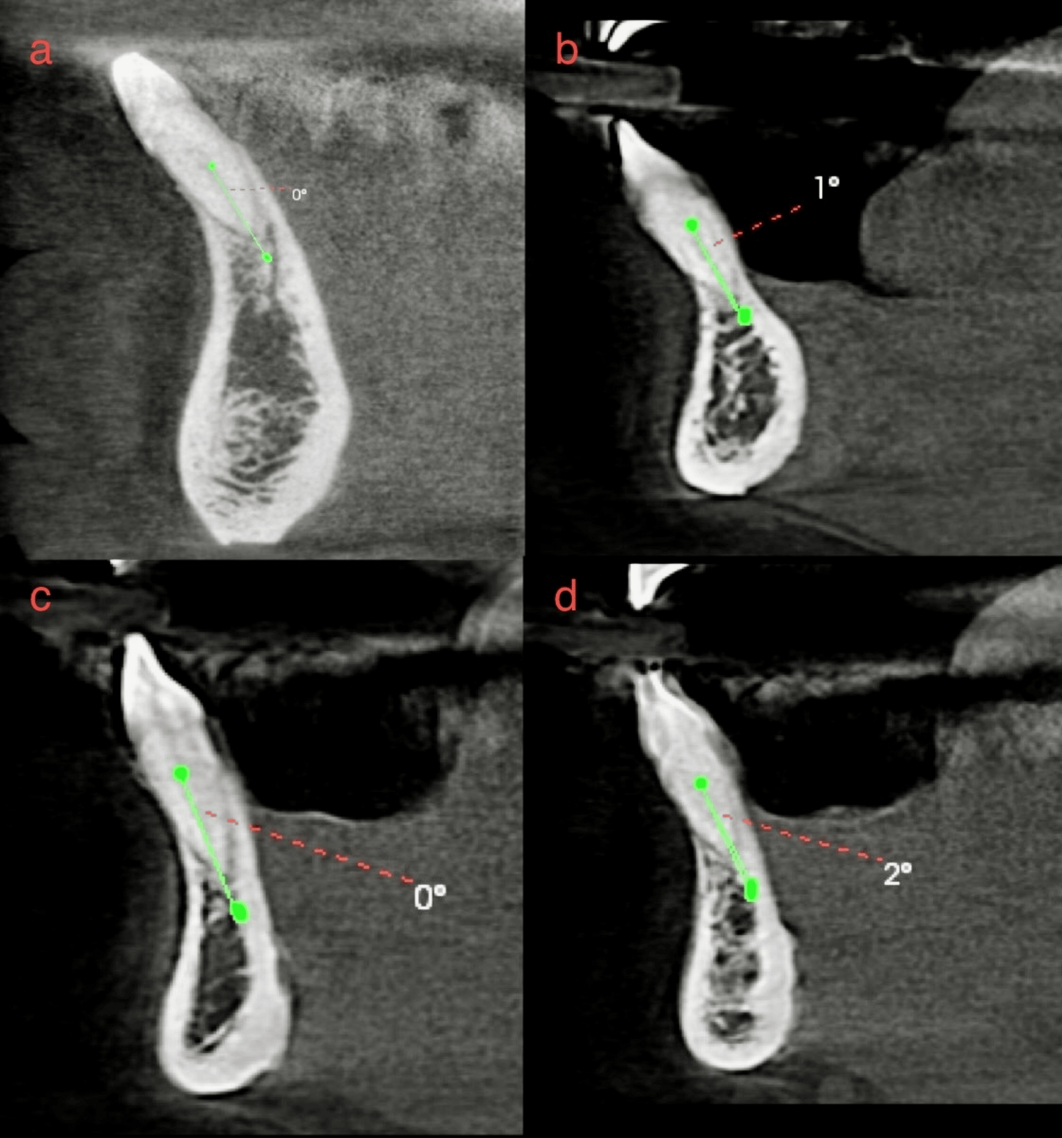
(a-d) Four different CBCTs in which the external root curvature was determined using Schneider's Classification and found to be straight as the angle was <5 degrees. CBCT - Cone beam computed tomography

**Figure 3:**
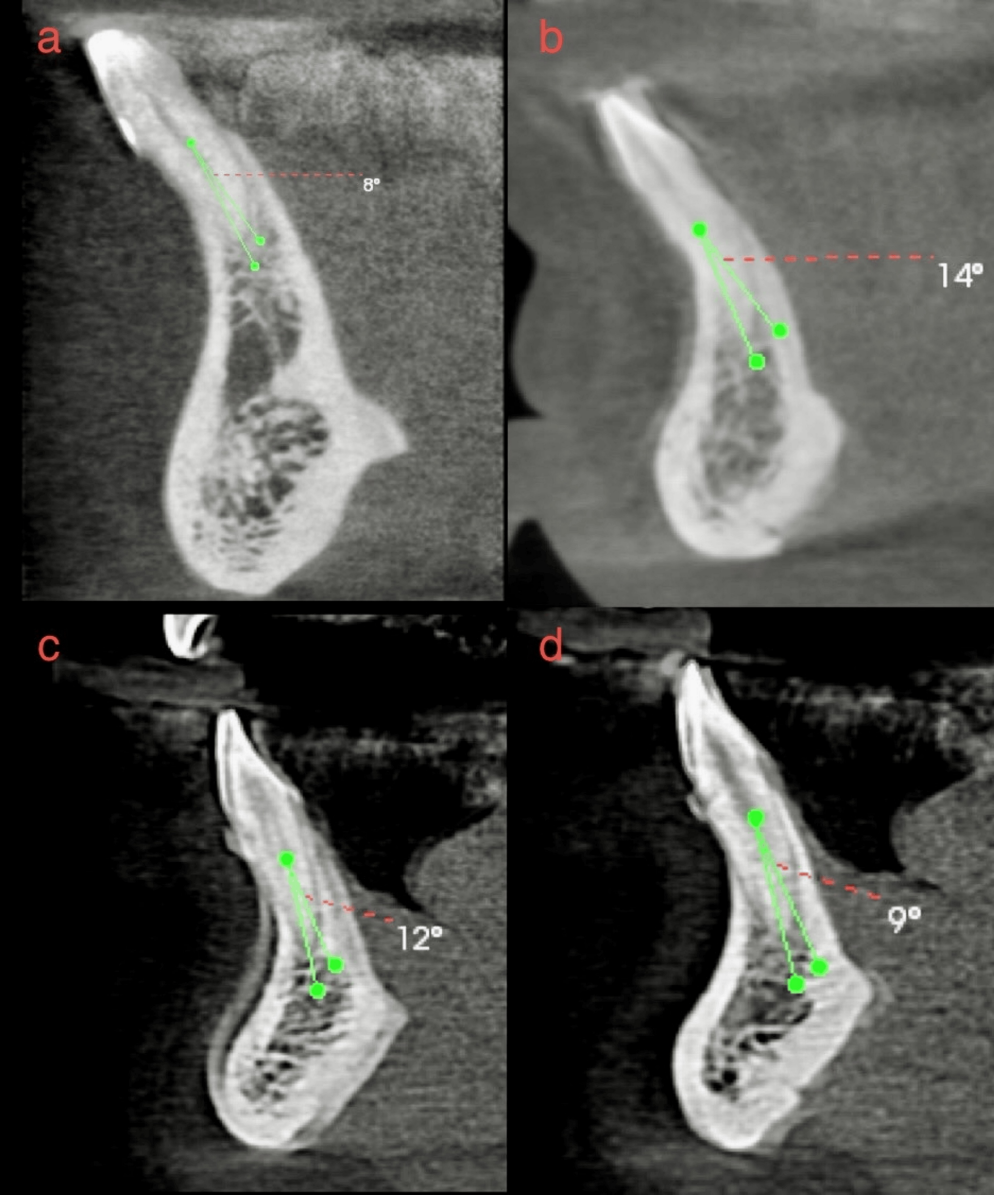
(a-d) Four different CBCTs in which the external root curvature was determined using Schneider's Classification and found to be curved as the angle was >5 degrees. CBCT - Cone beam computed tomography

**Figure 4:**
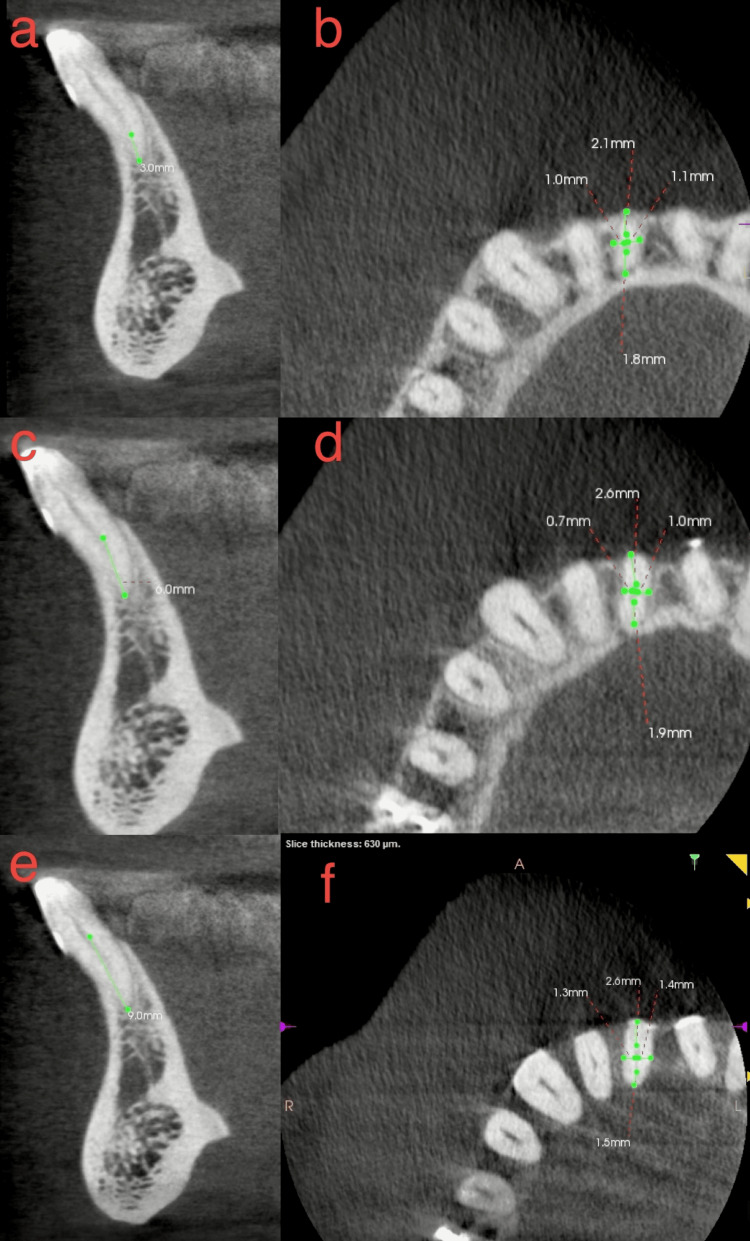
Multiple CBCT depicting measurement of root dentin thickness. (a, c, e) Measurement of root level at 3, 6, 9 mm from the apex, respectively. (b, d, f) Measurement of dentin labially, lingually, distally, and mesially at 3, 6, 9 mm from the apex. CBCT - Cone beam computed tomography

Images obtained were observed for external morphology such as the number of roots, mean root length (length from root apex to Cementoenamel junction) (Figures 1a-1d) external root curvature (based on Schneider Classification), and the direction of curvature (Figures 2a-2d, 3a-3d). Internal morphology such as the number of canals, level of bifurcation of the canal, and canal configurations using Vertucci and Ahmed's classification system. DT and canal shape at 3, 6, 9 mm from the apex were also measured using the methodology followed by Espir et al. The canal shape was determined by finding the ratio of labiolingual and MD DT, which was termed the diameter ratio (DR) if DR>4, it was termed flattened; oval if 2≤DR≥4; rounded if 1.1<DR>2; and round if 0.9≤DR≥1.1) (Figures 4a-4d). The images of CBCTs with MI were assessed by two examiners previously calibrated using 40 CBCTs. One examiner was a Postgraduate student, and another examiner was an Endodontist with 17 years' experience. The Images were viewed on the same display by both examiners. Interexaminer agreement measured with Cohen’s kappa between the two examiners was calculated to be 0.66 with the percentage of agreement at 83.33% showing substantial agreement.

Statistical Package for Social Sciences (SPSS) for Windows (Version 22.0, Released 2013. IBM Corp., Armonk, NY) was used to perform statistical analyses. Descriptive analysis of all the explanatory and outcome parameters was done using mean and standard deviation for quantitative variables and frequency and proportions for categorical variables.

## Results

**Figure 5:**
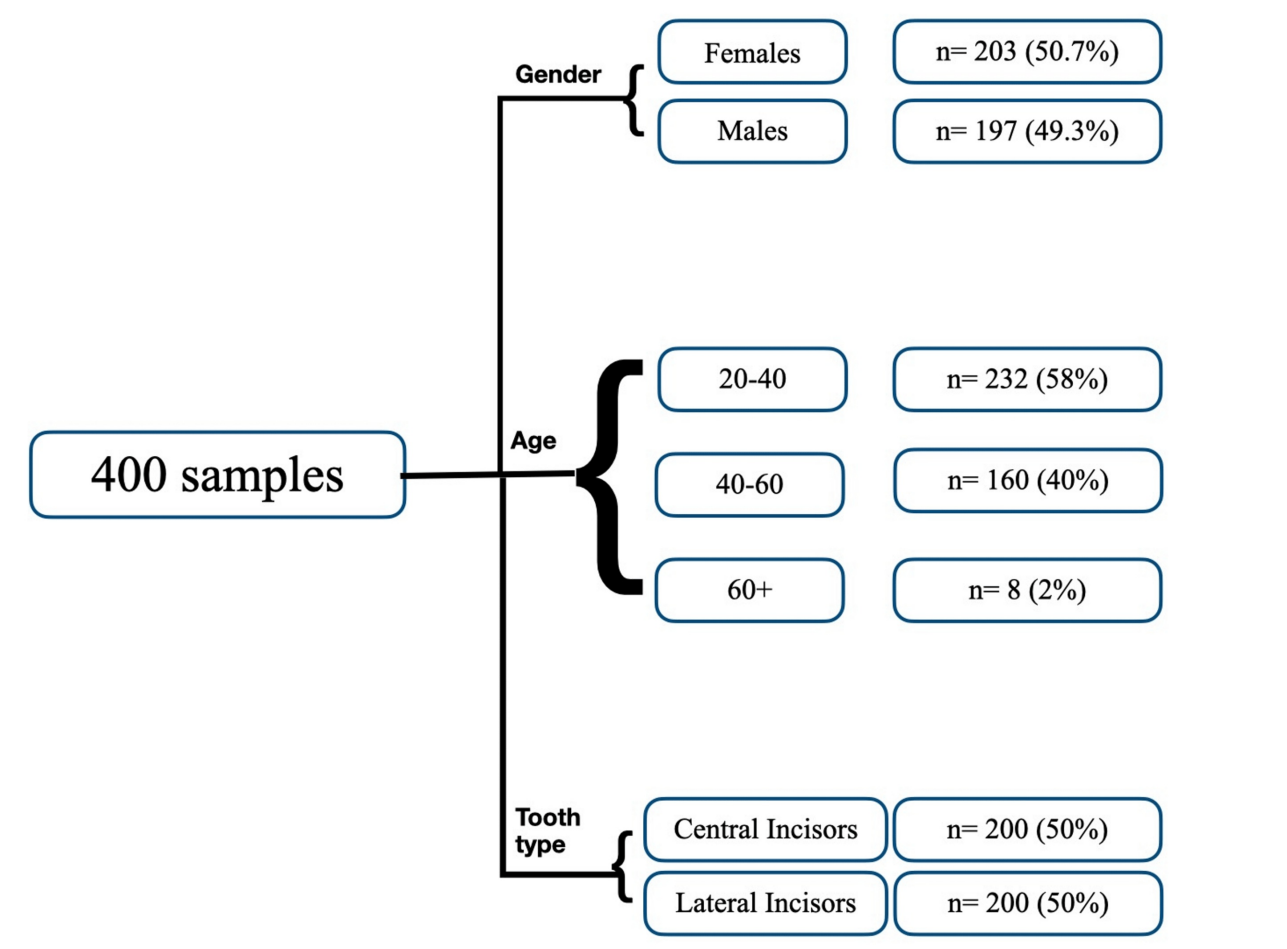
Flowchart depicting the distribution of samples based on age, gender, and tooth type

CBCT images with 400 MI were analyzed for this study. Among the analyzed samples, 50.7% (203) were females and 49.3% (197) were male participants. Patient age was divided into three groups: 20-40, 41-60 and 60+. Among the 400 samples, 200 were central incisors (CI) and 200 were lateral incisors (LI) (Figure 5).

When analyzing external morphology, all 200 CI and 200 LI had a single root. The CI had a mean root length of 12.769 ± 1.128 mm, while the LI had 13.044 ± 1.235 mm. Among the CI, 96% (192) had straight roots, and 4% (eight) had curved roots. Of the curved roots, 3.5% (seven) had distal curvature, and 0.5% (one) had buccal curvature. Among the LI, 95% (190) had straight roots, and 5% (10) had curved roots. Of the curved roots, 2.5% (five) had distal curvature, 2% (four) had buccal curvature, and 0.5% (one) had mesial curvature (Table 1).

**Table 1:** Distribution of samples based on number of roots, mean root length, and external root curvature in central and lateral incisor.

	Central incisor (N = 200)	Lateral incisor (N= 200)
1. No of roots	Percentage (%)	Percentage (%)
Single root	100	100
Two roots	0	0
2. Mean root length	In mm	In mm
	12.769 ± 1.128	13.044 ± 1.235 mm
3. Root curvature	Percentage (%)	Percentage (%)
Straight	96	95
Distally curved	3.5	2.5
Buccally curved	0.5	2
Mesially curved	-	0.5

**Figure 6:**
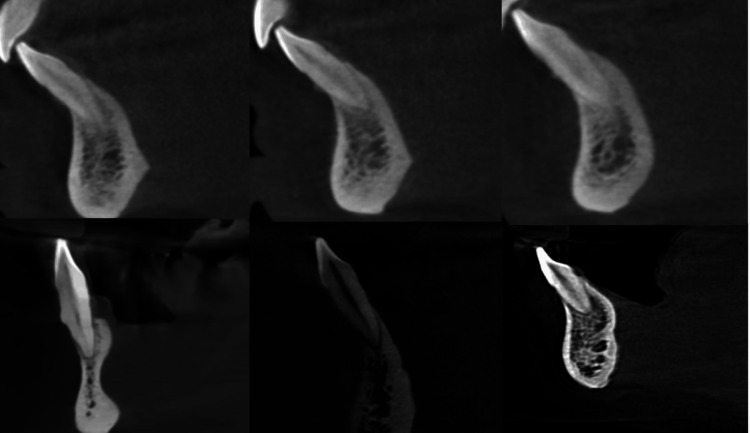
Multiple CBCTs depicting single canal or type 1 Vertucci configuration. CBCT - Cone beam computed tomography

**Figure 7:**
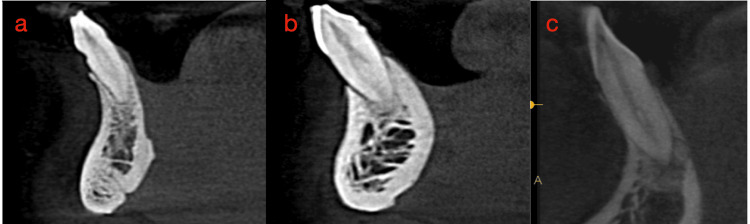
CBCTs depicting two canals and bifurcations of canals. (a) Bifurcation at coronal third. (b) Bifurcation at middle third. (c) Bifurcation at apical third. CBCT - Cone beam computed tomography

**Figure 8:**
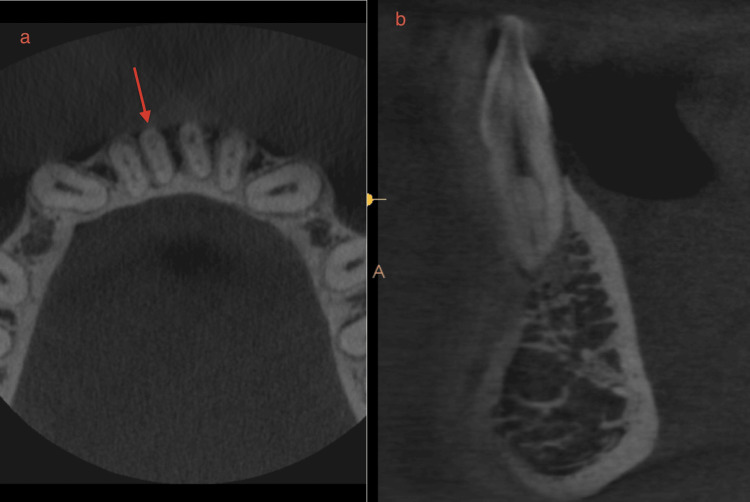
CBCT showing three canals or Ahmed 1-3-1 configuration. (a) Axial section. (b) Sagittal section. CBCT - Cone beam computed tomography

When analyzing internal morphology, for CI, a single canal (Figure 6) was found in 63.5% (127), two canals (Figure 7) in 36% (72), and three canals (Figure 8) in 0.5% (one). Additionally, 63.5% (127) of CI had no bifurcations, 24.5% (49) had canals bifurcating at the middle third, 11.5% (23) bifurcated at the coronal third, and 0.5% (one) at the apical third (Figure 7). For LI, a single canal was found in 60.5% (121) and two canals in 39.5% (79), with no LI having three canals. Also, 60.5% (121) had no bifurcations, 26.5% (53) had canals bifurcating at the middle third, and 13% (26) bifurcating at the coronal third. When analyzing canal configurations using the Vertucci and Ahmed classifications for CI, the most common was type 1/^1^CI^1^ (Figure 6) at 63.5% (127), followed by type 3/^1^CI^1-2-1^ at 33% (66), type 5/^1^CI^1-2^ at 2% (four), and type 2/^1^CI^2-1^ at 1% (two). One tooth, not classifiable by Vertucci, was classified by Ahmed as ^1^CI^1-3-1^ (Figure 8). For LI, the most common was type 1/^1^LI^1^ at 60.5% (121), followed by type 3/^1^LI^1-2-1^ at 36% (72), type 5/^1^LI^1-2^ at 2% (four), type 2/^1^LI^2-1^ at 1% (one), and type 7/^1^LI^1-2-1-2 ^at 0.5% (one). The one tooth not classifiable by Vertucci, found in a female, was classified as ^1^CI^1-3-1 ^by Ahmed. Table 2 shows the distribution of samples based on the number of canals, level of bifurcations of the canal, and canal configurations.

**Table 2:** Distribution of samples based on the number of canals, level of bifurcations of the canal, and canal configurations.

	Central incisor (N = 200)	Lateral incisor (N = 200)
1. No. of canals	Percentage (%)	Percentage (%)
1	63.5	60.5
2	39	39.5
3	0.5	0
2. Level of bifurcation	Percentage (%)	Percentage (%)
No bifurcations	63.5	60.5
Apical	0.5	0
Middle	24.5	26.5
Coronal	11.5	13
3. Vertucci and Ahmed Classification, central incisor (CI), lateral incisor (LI)	Percentage (%)	Percentage (%)
Type 1 or ^1^CI^1^/^1^LI^1 ^	63.5	60.5
Type 2 or ^1^CI^2-1^/^1^LI^2-1^	1	1
Type 3 or ^1^CI^1-2-1^/^1^LI^1-2-1^	33	36
Type 5 or ^1^CI^1-2^/^1^LI^1-2^	2	2
Type7 or ^1^CI^1-2-1^/^1^LI^1-2-1-2^	-	0.5
^1^CI^1-3-1^	0.5	-

For CI, the analysis of DT and DR showed the following: At 3 mm from the apex, the mean LL was 3.18 ± 0.639 mm and the mean MD was 1.578 ± 0.342 mm. Among these, 58.5% (117) had oval-shaped canals and 41.5% (83) had rounded canals. At 6 mm from the apex, the mean LL was 3.72 ± 0.671 mm, and the mean MD was 1.881 ± 0.374 mm. Of these, 59% (118) had oval-shaped canals, 40.5% (81) had rounded canals, and 0.5% (one) had a round canal. At 9 mm from the apex, the mean LL was 4.43 ± 0.754 mm, and the mean MD was 2.283 ± 0.465 mm. Here, 44% (88) had oval-shaped canals and 56% (112) had rounded canals.

For LI, the analysis revealed that at 3 mm from the apex, the mean LL was 3.41 ± 0.916 mm, and the mean MD was 1.63 ± 0.322 mm. Among these, 61% (122) had oval-shaped canals, 38% (76) had rounded canals, and 1% (two) had a flattened canal. At 6 mm from the apex, the mean LL was 3.90 ± 0.702 mm, and the mean MD was 1.981 ± 0.485 mm. Of these, 40.5% (81) had oval-shaped canals, 59% (118) had rounded canals, and 0.5% (one) had a flattened canal. At 9 mm from the apex, the mean LL was 4.55 ± 0.746 mm, and the mean MD was 2.55 ± 0.470 mm. Here, 33% (66) had oval-shaped canals, 66% (132) had rounded canals, and 0.5% (one) had a round canal. Table 3 depicts root DT at 3, 6, and 9 mm from the apex in CI and LI. Table 4 depicts the distribution of canal shapes at 3, 6, and 9 mm from the apex in CI and LI.

**Table 3:** Root dentin thickness at 3, 6, and 9 mm from the apex in central and lateral incisors.

Tooth type	At 3 mm		At 6 mm		At 9 mm	
Labiolingual (LL) and mesiodistal (MD)	Mean LL distance	Mean MD distance	Mean LL distance	Mean MD distance	Mean LL distance	Mean MD distance
Central incisor (200)	3.182 ± 0.639	1.578 ± 0.342	3.72 ± 0.671	1.881 ± 0.374	4.43 ± 0.754	2.283 ± 0.465
Lateral incisors (200)	3.41 ± 0.916	1.63 ± 0.322	3.90 ± 0.702	1.98 ± 0.485	4.55 ± 0.746	2.55 ± 0.470
Total (400)	3.296 ± 0.916	3.82 ± 0.702	4.505 ± 0.746	1.604 ± 0.322	1.981 ± 0.485	2.422 ± 0.470

**Table 4:** Distribution of canal shapes at 3, 6, and 9 mm from apex in central and lateral incisors.

Tooth type	Level of root	Canal shape	Percentage
Central incisor	3 mm	Flattened	0
		Oval	58.5
		Rounded	41.5
		Round	0
	6 mm	Flattened	0
		Oval	59
		Rounded	40.5
		Round	0.5
	9 mm	Flattened	0
		Oval	44
		Rounded	56
		Round	0
Lateral incisor	3 mm	Flattened	1
		Oval	61
		Rounded	38
		Round	0
	6 mm	Flattened	0.5
		Oval	40.5
		Rounded	59
		Round	0
	9 mm	Flattened	0
		Oval	33
		Rounded	66
		Round	1

## Discussion

Mashyakhy et al. [10] reported that 90% of teeth with a missed canal can progress to apical periodontitis, underscoring the importance of understanding dental variations. Traditionally, MI has been treated as having a single canal. However, recent studies [3,7-9] have shown an increased prevalence of two canals ranging from 0.4% to 45% in different populations. Specifically, in the German population, the prevalence of two canals was 22.6% for CI and 24.3% for LI [11], In the Polish population, it was 34.1% for CI and 31.8% for LI [12]; in Saudi Arabia, the prevalence was 26.35% for CI and 30.8% for LI [13]; in Iran, it was 15.5% for CI and 21.8% for LI [14]. In Malaysia, it was 5.1% for CI and 12.3% for LI [15]. In Italy, it was 45% for CI and 43% for LI [16]. In China, it was 15.7% for CI and 27.4% for LI [17]. In Brazil, it was 35.5% for CI and 39.5% for LI [18]. Among the Indian population, various studies have reported different percentages of two canal occurrences: 8.34% to 31.8% for CI and 10.45% to 35% for LI [19].

As highlighted by Assadian et al. [20], CBCT offers significant advantages in appreciating missed canals and minor variations in tooth morphology compared to other methods. These advantages include high resolution, 3D visualization, minimal image distortion, and lower radiation exposure than conventional CT scans [14]. Methods, such as periapical radiographs, tooth sectioning and clearing, and micro-CT, are also used to study root morphology. Still, they are more invasive and require extracted teeth, which can be anatomically compromised and not representative of normal teeth [21]. Therefore, CBCT, being non-invasive, provides a compelling advantage over micro-CT in these cases.

The inclusion criteria for this study were chosen based on the understanding that root completion in MI teeth typically occurs around 11 years of age [22]. All samples analyzed in this study, both CI and LI had a single root. Previous studies [23] on root canal curvature have consistently found that straight canals are the most common type, which aligns with the findings of this study. Although the percentage of curved roots was slightly higher in CI than in LI, this difference was not statistically significant. Aminsobhani et al. [24] also reported similar findings, noting that 68.3% of roots had a straight orientation, while 15.1% were distally oriented and 5.2% were buccally oriented. This study observed a similar pattern, with straight roots being the most frequent, followed by distal, buccal, and mesial orientations of root curvature.

The mean root length was 12.769 ± 1.128 mm for CI and 13.044 ± 1.235 mm for LI in this study. This indicates that LI tends to have a slightly longer mean root length than CI, although this difference was not statistically significant. These results are consistent with previous studies reporting root lengths of 12.9 mm and 12.8 mm in CI and LI, respectively, in the Indian population [25]. Understanding root length is clinically important as it helps in determining the working length, which is critical in endodontic therapy.

The number of root canals observed varied between one, two, or three in this study, with the majority of samples having a single canal. Notably, 36% CI and 39.5% LI had two canals, which aligns with findings in the literature where the presence of a second canal ranges widely from 0.4% to 45% across different populations [3]. Previous studies consistently indicate that LI are more predisposed to having a second canal compared to CI [2,11,15], which was also found in this study. However, some studies, such as those by Sroczyk et al. [12] and Valenti-Obino et al. [16], have reported a higher percentage of second canals in CI, possibly due to racial and genetic differences among the studied populations. An interesting finding in this study was the presence of a third canal observed in one CI, while the remaining MI in the same patient had only two canals. The presence of a third canal is seldom reported in the literature [26] indicating its rarity across populations. Martins et al. [27] noted that the main root canal can merge and split at any level in the root, with this study confirming that bifurcations were most frequent in the middle third, followed by the coronal third, and rarely in the apical third. These findings corroborate with previous research by Boruah et al. [2] and Taha et al. [7], which consistently show a higher frequency of canal bifurcation in the middle and coronal thirds of the root.

The mean LL and MD dimensions and DT were observed to increase from the apical to the coronal sections in both CI and LI. This pattern is likely due to the conical shape of MI roots. While DT was slightly higher in LI than CI, this difference was not statistically significant. Additionally, DT tended to be greater LL than MD, consistent with findings by Espir et al. [4]. Silva et al. [28] suggested vertical root fractures are more common when the remaining DT is less than 1.3 mm. In this study, the mean MD thickness was observed to be 1.5 mm and 1.6 mm in CI, and 1.8 mm and 1.9 mm in LI, at the apical and middle thirds, respectively. These measurements emphasize the importance of avoiding aggressive canal preparation to preserve DT and reduce the risk of VRFs during endodontic procedures.

This study found that at 9 mm from the apex, most teeth had rounded canals. As the analysis moved closer to the apex, most canals became oval-shaped, with a higher percentage of oval-shaped canals in all three sections of the roots. Oval-shaped canals require modified root canal treatment, including instrumentation and obturation. Mohammadi et al. [29] advised using circumferential files in a reciprocating handpiece, non-square cross-sectional files, and thermoplastic obturation instead of lateral condensation for oval-shaped canals.

In this study, two systems of classifications, Vertucci and Ahmed, were utilized to assess canal configurations. The most frequent configuration observed was Vertucci type I, corresponding to Ahmed's ^1^CI^1^ and ^1^LI^1^. This was followed by type III, corresponding to ^1^CI^1-2-1^ and ^1^LI^1-2-1^, and type V, corresponding to ^1^CI^2-1^ and ^1^LI^2-1^. These findings align with previous studies by Iqbal et al. [8], Sroczyk-Jaszczyńska et al. [12], and Verma et al. [19]. Contrastingly, Karobari et al. [30] found in their study that type III or ^1^MI^1-2-1^ had the highest frequency, followed by type I or 1MI1. Additionally, this study identified other configurations such as type II Vertucci or ^1^CI^2-1 ^and ^1^LI^2-1^, and type VII Vertucci or ^1^LI^1-2-1-2^. Similarly, Baxter et al. [14] reported type II or 1CI2-1 and 1LI2-1 occurrences in 21.7% of samples, and Sroczyk et al. [12] reported type VII or ^1^MI^1-2-1-2^. One unique finding in this study was a configuration that could not be categorized under the Vertucci classification, classified as ^1^CI^1-3-1^ using Ahmed's classification. This highlights Ahmed's classification's capability to categorize various canal configurations effectively. While Vertucci classification is widely used, it has limitations such as the use of Roman numerals, which can be difficult to remember, and it does not depict roots in cases where teeth have multiple roots. In contrast, Ahmed's classification offers clarity and ease of communication among clinicians. Therefore, adopting Ahmed's classification in clinical practice could reduce ambiguity and enhance communication regarding root canal configurations.

Limitations of the study include that a very small sample size was analyzed and the geographical area from where the images were collected was limited. Future perspectives for this study may be done by assessing the relationship between ipsilateral and contralateral MI and the use of micro-CT, nano CT, and OCT to assess root canal morphology and variations.

## Conclusions

All 400 MI had a single root with an average length of 12.769 ± 1.128 in the CI and 13.044 ± 1.1235 in the LI, most of which were straight. Most samples had one canal in both CI and LI, followed by two canals in 36% CI and 39.5% LI. Canal bifurcations most frequently occurred in the middle third. Type 1/^1^MI^1 ^was the most common canal configuration, followed by type 3/^1^MI^1-2-1^, type 5/^1^MI^1-2^, and type 2/^1^MI^2^. One sample had a unique classification under Ahmed as ^1^CI^1-3-1^. The mean DT decreased, and canal shapes changed from round to oval when analyzed from coronal to apical third.

In the Indian population, MIs should not be assumed to have just a single canal; instead, clinicians should remain alert to the possibility of two or even three canals, often with bifurcations. Additionally, it is crucial to keep in mind the limited radicular DT during tooth preparation.
